# Diversity pattern of Duffy binding protein sequence among Duffy-negatives and Duffy-positives in Sudan

**DOI:** 10.1186/s12936-018-2425-z

**Published:** 2018-08-17

**Authors:** Mohammad Rafiul Hoque, Mohammed Mohieldien Abbas Elfaki, Md Atique Ahmed, Seong-Kyun Lee, Fauzi Muh, Musab M. Ali Albsheer, Muzamil Mahdi Abdel Hamid, Eun-Taek Han

**Affiliations:** 1 Department of Medical Environmental Biology and Tropical Medicine, School of Medicine, Kangwon National University, Chuncheon, Gangwon-do Republic of Korea; 2 Department of Parasitology and Medical Entomology, Institute of Endemic Diseases, University of Khartoum, Qassr Street, Khartoum, Sudan; 3 Department of Microbiology and Parasitology, Faculty of Medicine, Jazan University, Jizan, Saudi Arabia

**Keywords:** Malaria, *Plasmodium vivax*, Sudan, Haplotype, Genetic diversity

## Abstract

**Background:**

Vivax malaria is a leading public health concern worldwide. Due to the high prevalence of Duffy-negative blood group population, *Plasmodium vivax* in Africa historically is less attributable and remains a neglected disease. The interaction between Duffy binding protein and its cognate receptor, Duffy antigen receptor for chemokine plays a key role in the invasion of red blood cells and serves as a novel vaccine candidate against *P. vivax*. However, the polymorphic nature of *P. vivax* Duffy binding protein (DBP), particularly N-terminal cysteine-rich region (PvDBPII), represents a major obstacle for the successful design of a DBP-based vaccine to enable global protection. In this study, the level of *pvdbpII* sequence variations, Duffy blood group genotypes, number of haplotypes circulating, and the natural selection at *pvdbpII* in Sudan isolates were analysed and the implication in terms of DBP-based vaccine design was discussed.

**Methods:**

Forty-two *P. vivax*-infected blood samples were collected from patients from different areas of Sudan during 2014–2016. For Duffy blood group genotyping, the fragment that indicates GATA-1 transcription factor binding site of the *FY* gene (− 33T > C) was amplified by PCR and sequenced by direct sequencing. The region II flanking *pvdbpII* was PCR amplified and sequenced by direct sequencing. The genetic diversity and natural selection of *pvdbpII* were done using DnaSP ver 5.0 and MEGA ver 5.0 programs. Based on predominant, non-synonymous, single nucleotide polymorphisms (SNPs), prevalence of Sudanese haplotypes was assessed in global isolates.

**Results:**

Twenty SNPs (14 non-synonymous and 6 synonymous) were identified in *pvdbpII* among the 42 Sudan *P. vivax* isolates. Sequence analysis revealed that 11 different PvDBP haplotypes exist in Sudan *P. vivax* isolates and the region has evolved under positive selection. Among the identified PvDBP haplotypes five PvDBP haplotypes were shared among Duffy-negative as well as Duffy-positive individuals. The high selective pressure was mainly found on the known B cell epitopes (H3) of *pvdbpII*. Comparison of Sudanese haplotypes, based on 10 predominant non-synonymous SNPs with 10 malaria-endemic countries, demonstrated that Sudanese haplotypes were prevalent in most endemic countries.

**Conclusion:**

This is the first *pvdbp* genetic diversity study from an African country. Sudanese isolates display high haplotype diversity and the gene is under selective pressure. Haplotype analysis indicated that Sudanese haplotypes are a representative sample of the global population. However, studies with a large number of samples are needed. These findings would be valuable for the development of PvDBP-based malaria vaccine.

**Electronic supplementary material:**

The online version of this article (10.1186/s12936-018-2425-z) contains supplementary material, which is available to authorized users.

## Background

Vivax malaria is a leading public health concern worldwide [1]. Out of 6 malaria parasites that infect humans, *Plasmodium vivax* is the second lethal cause of infection after *Plasmodium falciparum*, which is widely distributed in malaria-endemic regions where approximately 2.85 billion people are at risk [2]. Although global malaria cases are on the decline over the years, with the emergence of drug-resistant *P. vivax* strains and associated severe and fatal consequences and the ability to produce hypnozoites causing relapse in patients, there are clear indications of notable public health significance [3–7]. Eradication and control interventions targeting *P. falciparum* are supposed to not be fully effective against *P. vivax* which is certainly a major challenge for malaria eradication [8]. Therefore, there is an urgent need for a vaccine against this deadly parasite [9].

*Plasmodium vivax* invasion is primarily dependent on the interaction between Duffy binding protein and its corresponding receptor Duffy antigen receptor for chemokines (DARC) [10]. Recently there are confirmed *P. vivax* infections among Duffy-negative population, indicating alternate invasion pathways [11–13]. Therefore, *P. vivax* Duffy binding protein (PvDBP) is a novel vaccine candidate because it induces strong immune responses in humans, and anti-DBP antibodies inhibit DBP–DARC interaction in vitro and also block merozoite invasion of human erythrocytes [14].

The erythrocyte-binding motif of DBP resides in the N-terminal cysteine-rich domain (C1–C12) known as DBPII [15]. However, the critical binding motif is a 170 amino acid (291–460 aa) stretch within the 330 aa of DBPII, which includes cysteines C4–C7 [16]. Successful invasion engages dimerization between 2 DBP and 2 DARC molecules [17]. Similar to other blood stage adhesion ligands, DBPII resides under strong immune selective pressure [18]. *PvdbpII* region is highly polymorphic with 4 times higher substitution rate compared to the rest of the gene, and polymorphisms at certain residues were found to be consistent in global *pvdbpII* isolates, demonstrating that polymorphisms help in immune evasion mechanism [19, 20]. Although such polymorphism is not known to affect the host-parasite binding, anti PvDBP antibodies, detected in residents of malaria-endemic areas, recognize PvDBPII but reveal significant differences regarding inhibitory responses [18].

The variable nature of the PvDBP, in particular DBPII, is a major challenge to the development of a vaccine against vivax malaria, restricting strain-transcending global protection [21]. The haplotype analysis using global *pvdbpII* sequences previously identified 7 haplotypes that can cover 60% parasite population in 8 malaria-endemic countries [22]. Such knowledge is important for the rational design of DBP-based vaccine against vivax malaria. However, existing data lack the information of DBP sequence polymorphism originating from an African country such as Sudan, as African *P. vivax* studies are neglected because of the high burden of *P. falciparum* cases and a high percentage of Duffy-negative population, which is usually thought to serve as a selective barrier to *P. vivax* infection [23]. Moreover, several studies revealed that *P. vivax* infection in African countries is not as low as was considered before [24]. High numbers of imported cases of *P. vivax* have also been documented in Chinese workers returning from African countries [25, 26]. Additionally, around 86.6 million Duffy-positive individuals were at risk in sub-Saharan countries; more attention to *P. vivax* in Africa is needed to control this parasite [24]. A recent study showed a 35.6% *P. vivax* infection rate in Central Sudan, indicating great public health significance of this neglected parasite [27]. However, there is no study of DBP sequence polymorphism from Sudan.

This study was conducted to determine the haplotype diversity of *pvdbpII* and Duffy blood group genotyping, natural selection of Sudanese *pvdbpII* and the haplotype prevalence based on predominant, non-synonymous single nucleotide polymorphisms (SNPs) in other endemic countries worldwide. The implication in terms of a DBP-based vaccine design is discussed.

## Methods

### Blood sample collection and DNA preparation and species identification by PCR

Sixty-three blood spots in Whatman filter papers were collected from cross-sectional surveys between 2014 and 2016 during the malaria transmission season (August to February) from malaria patients attending hospitals or health facilities in Sudan (Additional file 1). Initially, all samples were screened by rapid diagnostic kit (RDT) and microscopy for *P. vivax* infection. Genomic DNA was extracted using a QIAamp Blood Mini Kit Qiagen, Valencia, CA, USA) and kept at − 20 °C until used. Then *Plasmodium* species were identified by *18SrRNA* gene-based nested PCR using genus and species-specific primers as described previously [28].

### Duffy genotyping

A 252 bp fragment, which indicates GATA-1 transcription factor binding site of the *FY* gene (− 33T > C), was amplified using nested PCR. The primers were used as follows: Nest 1 Duffy_F: 5′-GGATGGAGGAGCAGTGAGAG-3′ and Nest 1 Duffy_R: 5′-CAAAGGGAGGGACACAAGAG-3′; Nest 2 Duffy_F: 5′-TAGTCCCAACCAGCCAAATC-3′ and Nest 2 Duffy_R: 5′-TCACCCTGTGCAGACAGTTC-3′. PCR amplification was performed using an Accupower premix (Bioneer, Daejeon, Korea) in a final volume of 20 µL containing 200 pM of each primer, 250 µM of each deoxyribonucleoside triphosphate, 1.5 mM MgCl_2_, 10 mM Tris-hydrochloric acid (pH 9.0), 30 mM KCl, 1.0 units of *Taq* polymerase and 2 µL of genomic DNA template. For nest 1 PCR reaction, the cycling conditions were: initial denaturing at 94 °C for 5 min followed by 35 cycles of 94 °C for 30 s, 59 °C for 1 min, and 72 °C for 45 s, and a final extension step of 10 min at 72 °C. The nest 2 primers were added with the nest 1 amplicons and amplified using the similar conditions except that the extension time of 25 s at 72 °C during 35 cycles. PCR products were analysed by electrophoresis on 1% agarose gel stained with 0.05% Redsafe dye (iNtRON Biotechnology, Daejeon, Korea). Amplicons were gel purified using DNA purification kit (Macherey–Nagel, Germany) as per manufacturer’s instruction. Purified products were sequenced in two directions using Nest 2 Duffy_F and Nest 2 Duffy_R primers by commercial sequencing company (Genotech, Daejeon, Korea) for genotyping. Sequences were aligned with NCBI reference Sequence: NG_011626.3 to detect a polymorphism (− 33T → C) for genotype confirmation.

### PCR amplification and sequencing

The region flanking *P. vivax dbpII* fragment (nucleotide positions 586–1848 bp; aa 196–617) was amplified using nested PCR. Nest 1 forward and reverse primers were Nest 1_F: GATAAAACTGGGGAGGAAAAAGAT and Nest 1_R: CCCGTAACAGCTTTACCTG, respectively. Nest 1 PCR amplification was performed using an Accupower premix (Bioneer, Daejeon, Korea) in a final volume of 20 µL containing 200 pM of each primer, 250 µM of each deoxyribonucleoside triphosphate, 1.5 mM MgCl_2_, 10 mM Tris-hydrochloric acid (pH 9.0), 30 mM KCl, 1.0 units of *Taq* polymerase and 3.5 µL of genomic DNA template. PCR cycling conditions were performed as follows: initial denaturing at 94 °C for 5 min followed by 35 cycles of 94 °C for 30 s, 59.6 °C for 30 s, and 72 °C for 1 min 30 s, and a final extension step of 10 min at 72 °C. Nest 2 forward and reverse primers were Nest 2_F: ATGTTAGATTATGAGACATCTAGCAA and Nest 2_R: AACAGCTTTACCTGTGGTAGAAC, respectively. Then nest 2 PCR amplification was performed using the similar reaction condition to nest 1 with the exception of 4 µL of nest 1 amplicon as template and thermal profile were used as follows; initial denaturing at 94 °C for 5 min followed by 35 cycles at 94 °C for 30 s, 57.6 °C for 30 s, and 72 °C for 1 min 30 s, and a final extension step of 10 min at 72 °C. PCR products were analysed by electrophoresis on 1% agarose gel stained with 0.05% Redsafe dye (iNtRON Biotechnology, Daejeon, Korea). Amplicons were gel purified using DNA purification kit (Macherey–Nagel, Germany) as per manufacturer’s instruction. Direct sequencing was performed using Nest 2_F and Nest 2_R by a commercial sequencing company (Genotech, Daejeon, Korea).

All sequences generated were assembled and edited using SeqMan and EditSeq, Lasergene v 7.0 (DNASTAR) and aligned using the Clustal W program in MegAlign Lasergene v 7.0 (DNASTAR). Samples having mixed genotype infections were excluded and any singleton site was confirmed by re-sequencing. SNPs at different residues were identified compared to Sal-1 (GenBank accession no. DQ156512) sequence as a reference. The sequences reported here have been deposited in the GenBank database under the accession numbers (MG805616–MG805657).

### DNA sequence and polymorphism analysis

DNA sequence polymorphism analysis was investigated on 42 Sudan *pvdbpII* sequences (nt. 727–1653). Using the DnaSP ver. 5.10.00 [32] following genetic diversity parameters were analysed; a number of segregating sites (S), nucleotide diversity (π), haplotype diversity (Hd), and the average number of pair-wise nucleotide differences within the population (k).

To explore the effect of overall natural selection in the fragment and on the epitope regions, rates of synonymous (dS) and non-synonymous (dN) substitutions was investigated using Nei and Gojobori method [29] with Jukes and Cantor correction. Tajima’s D test [30] and Fu and Li’s D and F tests [31] were performed with DnaSP ver. 5.10.00 [32]. For epitope regions, 10 known linear B-cell epitopes, which were classified into 3 distinct classes, high inhibitory (H), medium (M) inhibitory and low (L) inhibitory, were examined for natural selection [18].

### Analysis of predominant *pvdbpII* nucleotide haplotypes

In order to determine whether Sudanese haplotypes were prevalent in other endemic countries, 10 predominant non-synonymous SNPs were used, and these haplotypes were compared with haplotype data from 10 endemic countries (Mexico, Colombia, Brazil, Iran, India, Sri Lanka, Myanmar, Thailand, South Korea, Papua New Guinea).

### Sequences used in this study for analysis

Papua New Guinea (n = 113), (DQ156519; AF289480–AF289483; AF289635–AF289653 and AF291096; AY970837–AY970925, AF469515–AF469602); Colombia (n = 17), (U50575–U50590 and DQ156513); South Korea (n = 15), (DQ156515; DQ156522–DQ156523; AF215737–AF215738; AF220657, AF220659–AF220667); India (n = 100), (FJ491142–FJ491241); Thailand (n = 30), (EF219451, EF368159–EF368180, EF379127–EF379132, EF379134); Sri Lanka (n = 100), (GU143914–GU144013); Brazil (n = 123), (DQ156520, EU812839–EU812960); Iran (n = 11) (EU860428–EU860438); Myanmar (n = 12), (JN255576–JN255587); Mexico (n = 35), (KP759780–KP759814).

## Results

### Duffy genotypes

**Fig. 1:**
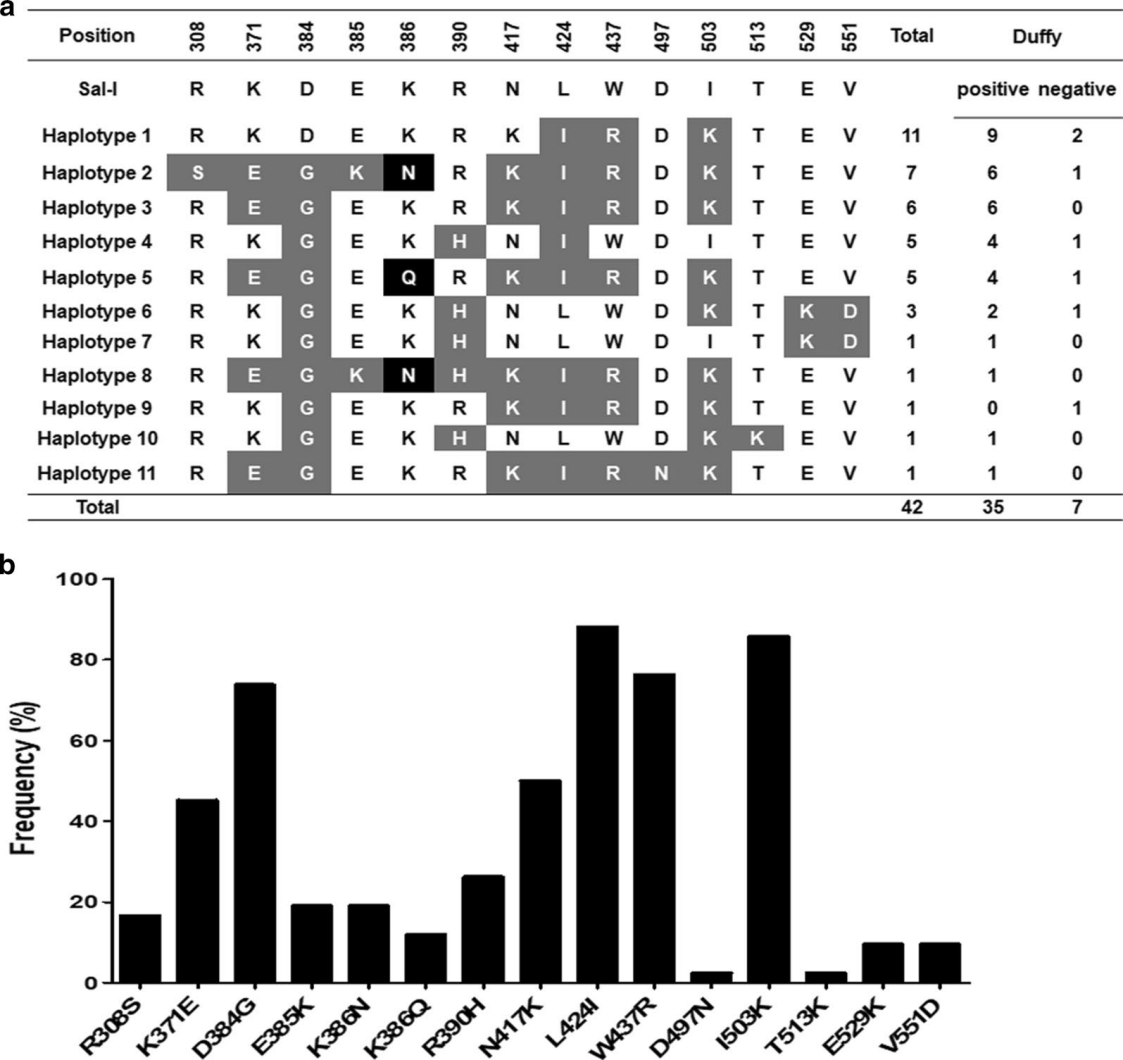
*PvdbpII* haplotypes and Duffy blood antigen genotypes observed among Sudanese isolates. **a** Polymorphic amino acid residues are listed for each haplotype. Amino acid residues identical to the reference sequence, Sal-1 (DQ156512), are marked with no color, dimorphic amino acid changes in grey and trimorphic in black. A total number of sequences for each *pvdbpII* haplotype and Duffy blood antigen genotype are listed in the right panel. **b** Frequencies of amino acid changes found in PvDBPII among Sudanese isolates

Duffy genotyping was performed to the isolates (n = 42) that could successfully amplify *pvdbpII* and generate high-quality sequences. Among those 42 *P. vivax*-infected patients, 35 (83.3%) were Duffy positive (Homologous 10 and Heterozygous 25) and 7 (16.7%) were Duffy negative (Fig. 1a).

### Genetic polymorphism of PvDBPII

Out of the 63 Sudanese isolates, the *pvdbpII* gene was successfully PCR amplified from 54 isolates. High-quality sequencing results for the PvDBPII fragments (927 bp) were obtained from 42 isolates, which were taken for further analysis. DNA sequence analysis of the 42 *pvdbpII* sequences showed that there was no size variation among the sequences compared to reference Sal-1 strain sequence. Twenty SNPs were found, 6 (30.0%) of which were synonymous and 14 (70.0%) were non-synonymous substitutions. Of these 20 SNPs, 7 occurred at the first base of the codon, 5 at the second, and 8 at the third base of the codon, resulting in significant amino acid changes in protein level (Fig. 1a). Among these, 2 singleton non-synonymous substitutions were detected (D497N and T513K). Five synonymous sites were found in 2.4% R408, 2.4% I380, 7.1% Q432, 2.4% A514, and 2.4% N531. Alignment of deduced amino acid sequences revealed 11 different haplotypes (haplotype 1–11) with amino acid changes at 14 positions in which one showed trimorphic polymorphism (K386N/Q) and the others were dimorphic (Fig. 1a). Duffy-negative *P. vivax* isolates reside in 6 different haplotypes out of 11 haplotypes (Fig. 1a). Of the 14 non-synonymous substitutions, 13 were reported in the previous study in the parasites circulating in different parts of the world, whereas one point mutation was found unique to current study (D497N, 2.4%,) [33–35]. All 12 cysteine residues were conserved among the 42 Sudanese isolates. Moreover, among all non-synonymous sites, frequency of residues K371E (47.6%), D384G (73.8%), N417K (76.2%), L424I (88.1%), W437R (76.2%), and I503K (85.7%) were higher than remaining sites (Fig. 1b). However, haplotype 1 was found dominant (26.2%) among other haplotypes (Fig. 1a). Comparison of the most common amino acid variants observed globally in PvDBPII revealed that Sudan isolates showed a comparatively similar pattern of minor allele frequency to Mexico isolates, but differed from other countries isolates (Papua New Guinea, Colombia, Brazil, Iran, India, Sri Lanka) (Table 1). Although Mexican isolates showed similar amino acid changes compared to Sudan isolates, 2 variants found in the Sudan isolates (D497N and T513K) were not identified in Mexican isolates [36].

**Table 1:** Minor allele frequencies of the most common PvDBPII amino acid variants

Isolate	PvDBPII variants forms on 10 non-synonymous position^a^									
	R308S	K371E	D384G	E385K	K386N	H390R	N417K	L424I	W437R	I503K
Sal-1	AGG	AAA	GAT	GAA	AAG	CGT	AAT	TTA	TGG	ATA
Variant	AG**T**	**G**AA	G**G**T	**A**AA	AA**T**	C**A**T	AA**A**	**A**TA	**C**GG	A**A**A
Country	Frequency									
Sudan	19.0	47.6	73.8	19.0	19.0	16.2	76.2	88.1	76.2	85.7
Mexico^b^	17.1	20.0	31.4	17.1	17.1	11.4	88.6	88.6	85.7	91.4
Colombia^c^	0.0	17.6	41.2	17.6	17.6	94.1	41.2	41.2	11.8	5.9
Brazil^c^	7.3	26.0	18.7	20.3	22.8	50.4	39.8	48.0	48.8	43.1
Myanmar^d^	22.2	22.2	85.2	33.3	33.3	63.0	38.9	83.3	61.1	77.8
Sri Lanka^e^	13.0	34.0	94.0	20.0	20.0	66.0	36.0	49.0	37.0	55.0
India^c^	10.0	33.0	87.0	31.0	31.0	65.0	38.0	45.0	38.0	47.0
Thai^f^	26.7	20.0	76.7	46.6	40.0	56.6	36.6	86.7	63.3	56.7
Korea^c^	0.0	46.7	46.7	6.7	6.7	53.3	93.3	100	100	100
Iran^g^	6.6	17.3	61.3	6.7	6.6	41.3	44.0	50.6	45.3	70.6
PNG^c^	69.0	11.5	34.5	9.7	9.7	50.4	33.6	68.1	32.7	42.5
Average	17.4	26.9	59.1	20.7	20.3	51.6	51.5	68.1	54.5	61.4

^a^The first letter represents the amino acid in that position in the Sal-1 sequence and the other letter represents the substituted amino acid; ^b^ [36]; ^c^ [22]; ^d^ [34]; ^e^ [35]; ^f^ [33]; ^g^ [42]

### Haplotype diversity and comparison with global populations

**Fig. 2:**
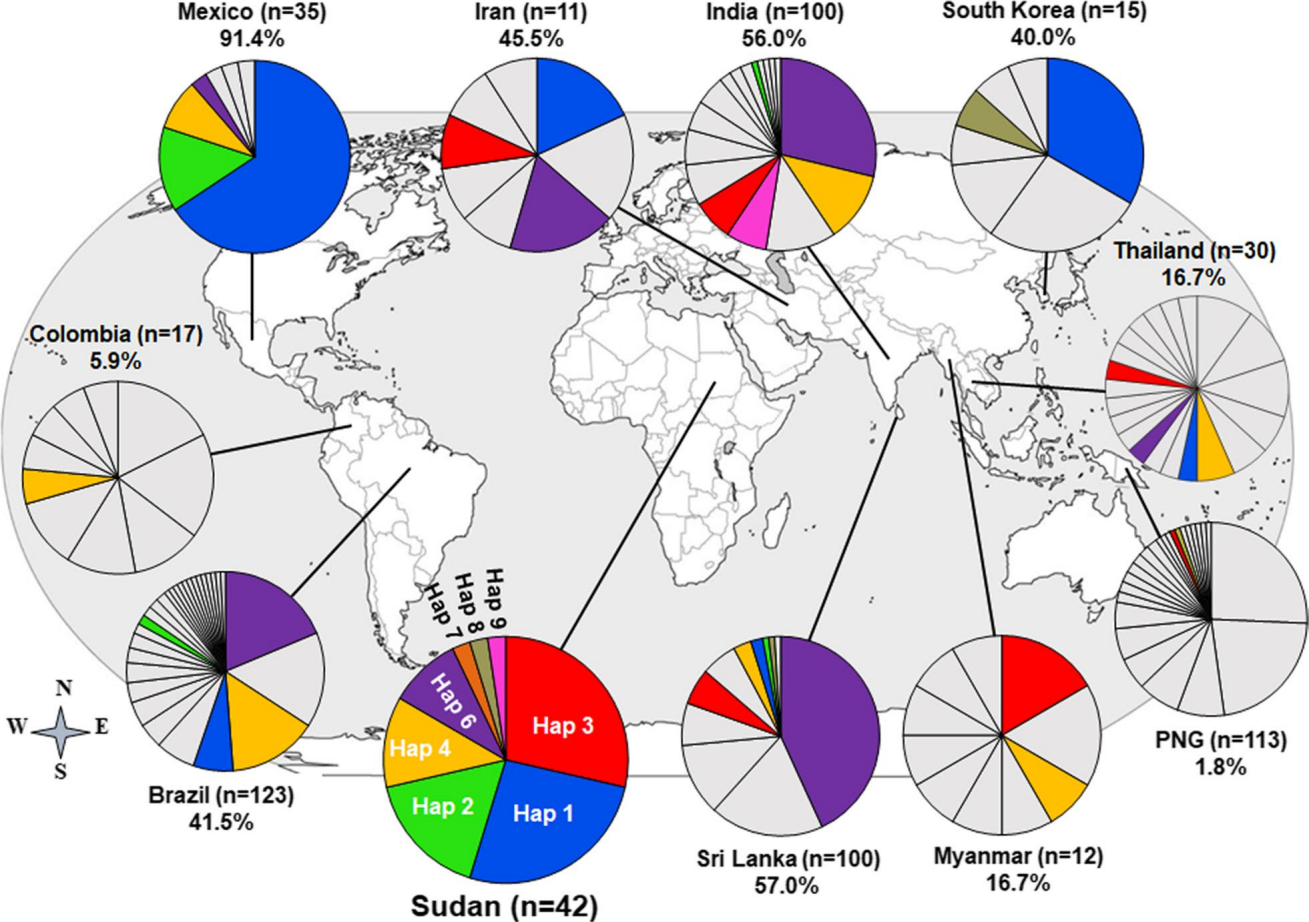
Worldwide prevalence of Sudanese *pvdbpII* haplotypes. The frequencies of 8 haplotypes based on 10 common non-synonymous polymorphic sites (Additional file 2) are depicted as pie charts and mapped to their geographic origin. Coloured segments indicate shared haplotypes with Sudan (dark blue = Haplotype 1, Sudan n = 11, Brazil n = 8, Iran n = 2, South Korea n = 5, Sri Lanka n = 2, Thai n = 1; bright green = Haplotype 2, Sudan n = 7, Brazil n = 2, Sri Lanka = 1, India n = 1; red = Haplotype 3, 5, 11 Sudan n = 12, Iran n = 1, PNG n = 1, Sri Lanka n = 6, Thai n = 1, India n = 7, Myanmar n = 1; Gold = Haplotype 4, Sudan n = 5, PNG n = 1, India n = 1; purple = Haplotype 6, 10 Sudan n = 4, Brazil n = 23, Iran n = 2, Sri Lanka n = 44, Thai n = 1, India n = 29; orange = Haplotype 7, Sudan n = 1, Brazil n = 18, Colombia n = 1, Sri Lanka n = 3, Thai n = 2, India n = 12, Myanmar n = 2; n = 1 and pink = Haplotype 8, Sudan n = 1, India n = 7; brown = Haplotype 9 Sudan n = 1 South Korea n = 1; Sri Lanka n = 1) and grey indicates other haplotypes to respective population. Note that haplotypes 5 and 11, and 10 correspond to haplotypes 3 and 6, respectively, because 10 non-synonymous SNPs out of 14 non-synonymous SNPs (Fig. 1a) were considered in this analysis

Based on the most common 10 non-synonymous SNPs observed globally, 8 haplotypes were found in Sudan (Additional file 2). The distribution of these haplotypes in 10 *P. vivax* endemic countries worldwide showed that all countries had at least one Sudanese haplotype, (Haplotype 4 in Colombia) and maximum 6 Haplotypes (Haplotype 1, 2, 3, 4, 6, and 9 in Sri Lanka) (Fig. 2) covering varied frequencies of parasite population, with the highest in Mexico (Haplotype 1, 2, 4 and 6, 91.4%) and lowest in Papua New Guinea (Haplotype 3 and 9, 1.8%) (Fig. 2). In a previous study, seven haplotypes were identified to cover an overall 60.0% worldwide parasite population but without any African countries [22]. Three of the haplotypes in the present study (Haplotype 1, 2 and 6) were shared with those haplotypes.

### Nucleotide diversity and natural selection of *pvdbpII*

**Fig. 3:**
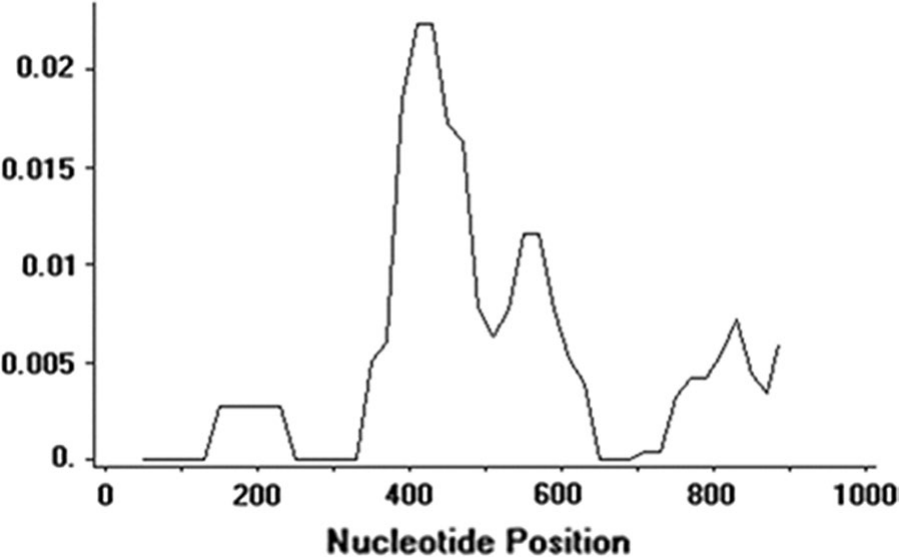
Graphical representation of *pvdbpII* nucleotide diversity (π). Sites with alignment gaps were not counted in the window length, window length: 100, and step size: 20

Analysis of molecular polymorphism within the 42 *pvdbpII* sequences revealed lower nucleotide diversity (π = 0.00497) and higher haplotype diversity (Hd = 0.900) which was similar to the diversity indices from several endemic countries [35]. Sliding window plot (window length 100 bp, step size 20 bp) using the DnaSP, revealed that nucleotide diversity was highest at 300–650 nt position of *pvdbpII* (Fig. 3). A significant positive natural selection (dN − dS = 2.05, p < 0.05) was found within the Sudanese *pvdbpII* sequences.

### Polymorphisms related to B- and T-cell epitopes

Analysis of natural selection in 10 known B-cell epitopes regions within Sudanese isolates indicated that the H3 epitope (residues 384–399 aa) was under positive selection (Table 2) whereas H2, M1, L1, L2, and L4 epitopes were conserved among the 42 Sudanese isolates (Table 2). The nucleotide diversity at epitope H3 was also high.

**Table 2:** Polymorphism analysis of inhibitory B-cell epitopes

Epitope^a^	Position (amino acid)	Epitope sequence (based on Sal-1)	S	Si	Pa	K	H	Hd ± SD	π ± SD	dN − dS	Taj D	D* (F&L)	F* (F&L)
H1	306–321	FH**R**DITFRKLYLKRKL	1	0	1	0.3	2	0.28 ± 0.077	0.00593 ± 0.001	0.007	0.31	0.55	0.56
H2	328–341	EGDLLLKLNNYRYN	0	0	0	0	0	0	0	0	0	0	0
H3	384–399	**DEK**AQQ**R**RKQWWNESK	5	0	5	1.6	6	0.81 ± 0.020	0.0341 ± 0.002	1.99^b^	1.68	1.11	1.27
M1	344–355	FCKDIRWSLGDF	0	0	0	0	0	0	0	0	0	0	0
M2	414–429	LKG**N**FIWICK**L**NVAVN	2	0	2	0.6	3	0.401 ± 0.085	0.012 ± 0.002	1.36	0.48	0.48	0.76
M 3	432–447	PQIYR**W**IREWGRDYVS	2	0	2	0.5	4	0.452 ± 0.078	0.010 ± 0.002	0.02	0.17	0.76	0.68
L2	400–411	AQIWTAMMYSVK	0	0	0	0	0	0	0	0	0	0	0
L3	364–377	MEGIGYS**K**VVENNL	1	0	1	0.5	2	0.511 ± 0.019	0.011 ± 0.0004	1.1	1.68	0.55	1.01
L1	282–293	CIPDRRYQLCMK	0	0	0	0	0	0	0	0	0	0	0
L4	272–286	DWDCNTKKDVCIPD	0	0	0	0	0	0	0	0	0	0	0

^a^[18]; ^b^ p < 0.02; S = number of variable (segregating) sites, Si = singleton variable sites, Pa = parsimony informative sites, K = average number of pair-wise nucleotide differences, H = number of haplotypes, Hd = haplotype diversity, SD = standard deviation, π = nucleotide diversity, dN = non synonymous substitution, dS = synonymous substitution, D⁄(F&L) = Fu and Li’s D⁄ test statistic, F⁄(F&L) = Fu and Li’s F⁄ test statistic. Bold and underline indicate polymorphic amino acid residues

## Discussion

*Plasmodium vivax*, the widely distributed malaria parasite is a great public health concern. A broadly effective malaria vaccine is a powerful tool for the control, elimination and eradication of this deadly disease [21]. Scientists across the globe are searching for a good strategy that provides strain transcending as well as long-term protection. However, the high degree of diversity of parasite antigens and the strain-specific immune response are challenging issues in the success of malaria vaccines [18]. In the current study, the genetic diversity and natural selection of DBPII have been investigated among Sudanese *P. vivax* isolates, and this is the first report of any African origin *P. vivax* isolates.

Based on amino acid variants, 11 different PvDBPII haplotypes were identified in the Sudanese isolates. Except for one unique haplotype (Haplotype 11), the majority of the haplotypes identified in this study were previously reported from other geographical areas (Mexico, Brazil, Iran, India, Sri Lanka, Thailand, South Korea) [22, 33–36]. Haplotype 1 was found to be the most prevalent in Sudanese isolates. Haplotype analysis showed that the number of identified haplotypes among Sudanese *P. vivax* isolates (26.2%) was higher than those previously reported from Mexico (22.9%) and Myanmar (22.2%) [34, 36], but it was lower than those reported from Sri Lankan (33.0%) and Brazilian (27.9%) isolates [35, 37].

Although Duffy negativity act as a selective barrier for *P. vivax* infection, few studies reported Duffy-negative *P. vivax* infection from several African countries [11–13]. Interestingly, in the current study, 7 Duffy-negative *P. vivax* cases were found, and they reside into six PvDBP haplotypes. However, it was found that 5 PvDBP haplotypes were shared among Duffy-negative as well as Duffy-positive individuals, except for one haplotype (Fig. 1a), which was unique to a Duffy-negative individual. Moreover, the 2 PvDBP Malagasy haplotypes from 2 Duffy-null individuals were also not identical to any of the haplotypes found in Sudan [38]. The findings of the shared PvDBP haplotypes between Duffy-positive and Duffy-negative individuals raise the question whether they have a role in *P. vivax* infection in Duffy-null individuals or not? The previous study reported that mutations in PvDBP did not relate to the ability of *P. vivax* to infect Duffy-null individuals [38]. An expansion of PvDBP copy number in Duffy-null *P. vivax* infection was documented which might allow binding the DBP with another ligand on Duffy-null erythrocyte [38]. Further PvDBP haplotype diversity, as well as a functional study with a large number of *P. vivax* isolates infecting Duffy-negative individuals will be needed to elucidate the exact function of shared haplotypes between Duffy-positive and Duffy-negative individuals and their role in *P. vivax* infection. Among the 14 non-synonymous mutations identified in the current study, 13 were previously reported, whereas the D497N was unique to Sudan isolates [33–37]. It was also found that all 12 cysteine residues were conserved, indicating the conserved binding ability to host erythrocytes in the field. However, previous studies revealed that these non-synonymous mutations do not affect the binding to the erythrocyte receptor but rather promote the parasite for immune evasion [18]. Three residues, N417K, W437R and I503K forming an important discontinuous epitope in PvDBP, were described as the main target for binding inhibitory antibodies against erythrocyte binding [39–41]. In the present study, N417K (76.2%), W437R (76.2%) and I503K (85.7%) were detected and these 3 SNPs were found simultaneously among 35 isolates (83.3%), which suggests a strong association between these residues in Sudanese *P. vivax* isolates. The frequency of these 3 SNPs was similar to that reported from Mexico but different from Sri Lanka, Colombia, Iran, and Myanmar [19, 34, 35, 42].

Many previous *pvdbpII* diversity studies documented high rates of non-synonymous mutations relative to synonymous mutations (dN − dS > 1.9) that reflected a positive natural selection promoting greater diversity of *pvdbpII*, probably to avoid host immune responses irrespective of the geographical distribution [18]. In this study, analysis of natural selection within the 42 Sudanese *P. vivax* isolates also identified significant positive selection (dN − dS = 2.05, p < 0.05). These results indicate that by increasing polymorphisms within the *pvdbpII*, parasites escape from the host immune response [18]. Natural selection in the known B-cell epitope regions in Sudanese isolates indicated that only H3 epitope (aa 384–399) was found to be under strong positive selection that could generate greater diversity. Higher genetic diversity was also found in the H3 epitope region. Tajima’s D, Fu and Li’s D^*^, and Fu and Li’s F^*^ for the H3 epitope region was positive but not significant. Similar strong selection pressure in this epitope region was observed in Sri Lankan and Iranian isolates [35, 42].

The number of migrant workers from *P. vivax*-endemic countries has risen in most African countries, including Sudan [27, 43]. Recently, *P. vivax* infections have been reported in high numbers in Sudan. This rising trend is possibly due to human migration from several Asian and African countries to work at newly formed petroleum and sugar companies in Sudan [27]. It was hypothesized that Sudanese *pvdbpII* haplotype might be a representative sample of global *P. vivax* population and this information could be used for the rational design of a PvDBP-based multi-allele vaccine. On this basis, haplotypes were generated considering 10 most frequent polymorphism sites of *pvdbpII* and 8 haplotypes were identified from Sudanese *P. vivax* isolates (Additional file 2). The prevalence of these 8 haplotypes was observed in all 10 malaria-endemic countries with a variable frequency that covers the highest 91.4% parasite population in Mexico and lowest in Papua New Guinea (1.8%) (Fig. 2), with an overall coverage of 36.8% (Fig. 2). However, haplotype analysis from seven endemic countries (Mexico, Brazil, Iran, India, Sri Lanka, Thailand, South Korea) indicated that haplotypes from Sudan covered up to 50% parasite populations. The current study was conducted using a low number of samples from Sudan (n = 42) and the information generated can be considered preliminary, yet it was representative of global *P. vivax* population and further studies with a large number of samples from Sudan is required to come to conclusions. In the current study among the identified haplotypes, none was identical to the Sal-1 isolate, which was also found absent in Thailand [22]. However, several studies from other malaria-endemic countries documented Sal-1 at low frequencies [22]. Although a phase 1 trial of PvDBP vaccine based on the Sal-1 sequence has been reported [44] it might be an inefficient strategy because it covers only 10% of the worldwide samples [22]. To cover the major portion of the *P. vivax* population multi-allele PvDBP might be a good approach. In a previous study, a similar multi-allele vaccine strategy with a minimum number of haplotypes using worldwide *pvdbpII* sequences identified seven haplotypes that covered an overall 60% worldwide parasite population but without any African countries [22]. In this study, out of 8 Sudanese haplotypes, 3 haplotypes were shared with that previous study those were (haplotypes 1, 6 and 7) suggested for multi-allele DBP vaccine approach.

## Conclusions

The findings provide the first description of genetic polymorphism and natural selection of *pvdbpII* in Sudan *P. vivax* isolates. Further investigations using a large number of isolates from different regions of Sudan and other African countries are needed to know the diversity of *pvdbp,* which is important to design and develop a DBP-based vaccine.

## Additional files

**Additional file 1.** Map of Sudan indicating the sample collection sites.

**Additional file 2.** Haplotypes based on 10 common polymorphism sites in Sudan isolates.

## References

[1] Mendis K, Sina BJ, Marchesini P, Carter R (2001). The neglected burden of *Plasmodium vivax* malaria. Am J Trop Med Hyg.

[2] Guerra CA, Howes RE, Patil AP, Gething PW, Van Boeckel TP, Temperley WH (2010). The international limits and population at risk of *Plasmodium vivax* transmission in 2009. PLOS Negl Trop Dis..

[3] Alexandre MA, Ferreira CO, Siqueira AM, Magalhaes BL, Mourao MP, Lacerda MV (2010). Severe *Plasmodium vivax* malaria, Brazilian Amazon. Emerg Infect Dis..

[4] Fernando D, Rodrigo C, Rajapakse S (2011). Primaquine in vivax malaria: an update and review on management issues. Malar J..

[5] Genton B, D’Acremont V, Rare L, Baea K, Reeder JC, Alpers MP (2008). *Plasmodium vivax* and mixed infections are associated with severe malaria in children: a prospective cohort study from Papua New Guinea. PLoS Med..

[6] Kochar DK, Das A, Kochar SK, Saxena V, Sirohi P, Garg S (2009). Severe *Plasmodium vivax* malaria: a report on serial cases from Bikaner in northwestern India. Am J Trop Med Hyg.

[7] Mohan K, Maithani M (2010). Congenital malaria due to chloroquine-resistant *Plasmodium vivax*: a case report. J Trop Pediatr.

[8] Mueller I, Galinski MR, Baird JK, Carlton JM, Kochar DK, Alonso PL (2009). Key gaps in the knowledge of *Plasmodium vivax*, a neglected human malaria parasite. Lancet Infect Dis..

[9] Mueller I, Shakri AR, Chitnis CE (2015). Development of vaccines for *Plasmodium vivax* malaria. Vaccine..

[10] Miller LH, Mason SJ, Clyde DF, McGinniss MH (1976). The resistance factor to *Plasmodium vivax* in blacks: the Duffy-blood-group genotype, FyFy. N Engl J Med..

[11] Menard D, Barnadas C, Bouchier C, Henry-Halldin C, Gray LR, Ratsimbasoa A (2010). *Plasmodium vivax* clinical malaria is commonly observed in Duffy-negative Malagasy people. Proc Natl Acad Sci USA.

[12] Ryan JR, Stoute JA, Amon J, Dunton RF, Mtalib R, Koros J (2006). Evidence for transmission of *Plasmodium vivax* among a Duffy antigen negative population in western Kenya. Am J Trop Med Hyg.

[13] Wurtz N, Lekweiry KM, Bogreau H, Pradines B, Rogier C, Boukhary AOMS (2011). Vivax malaria in Mauritania includes infection of a Duffy-negative individual. Malar J..

[14] Grimberg BT, Udomsangpetch R, Xainli J, McHenry A, Panichakul T, Sattabongkot J (2007). *Plasmodium vivax* invasion of human erythrocytes inhibited by antibodies directed against the Duffy binding protein. PLoS Med..

[15] Singh AP, Ozwara H, Kocken CH, Puri SK, Thomas AW, Chitnis CE (2005). Targeted deletion of *Plasmodium knowlesi* Duffy binding protein confirms its role in junction formation during invasion. Mol Microbiol.

[16] Ranjan A, Chitnis CE (1999). Mapping regions containing binding residues within functional domains of *Plasmodium vivax* and *Plasmodium knowlesi* erythrocyte-binding proteins. Proc Natl Acad Sci USA.

[17] Batchelor JD, Zahm JA, Tolia NH (2011). Dimerization of *Plasmodium vivax* DBP is induced upon receptor binding and drives recognition of DARC. Nat Struct Mol Biol.

[18] Chootong P, Ntumngia FB, VanBuskirk KM, Xainli J, Cole-Tobian JL, Campbell CO (2010). Mapping epitopes of the *Plasmodium vivax* Duffy binding protein with naturally acquired inhibitory antibodies. Infect Immun.

[19] Cole-Tobian J, King CL (2003). Diversity and natural selection in *Plasmodium vivax* Duffy binding protein gene. Mol Biochem Parasitol.

[20] Xainli J, Adams JH, King CL (2000). The erythrocyte binding motif of *Plasmodium vivax* Duffy binding protein is highly polymorphic and functionally conserved in isolates from Papua New Guinea. Mol Biochem Parasitol.

[21] Chen E, Salinas ND, Huang Y, Ntumngia F, Plasencia MD, Gross ML (2016). Broadly neutralizing epitopes in the *Plasmodium vivax* vaccine candidate Duffy Binding Protein. Proc Natl Acad Sci USA.

[22] de Sousa TN, Carvalho LH, de Brito CFA (2011). Worldwide genetic variability of the Duffy binding protein: insights into Plasmodium vivax vaccine development. PLoS One..

[23] Howes RE, Patil AP, Piel FB, Nyangiri OA, Kabaria CW, Gething PW (2011). The global distribution of the Duffy blood group. Nat Commun..

[24] Howes RE, Reiner RC, Battle KE, Longbottom J, Mappin B, Ordanovich D (2015). *Plasmodium vivax* transmission in Africa. PLOS Negl Trop Dis..

[25] Feng J, Xiao H, Zhang L, Yan H, Feng X, Fang W (2015). The *Plasmodium vivax* in China: decreased in local cases but increased imported cases from Southeast Asia and Africa. Sci Rep..

[26] Li Z, Yang Y, Xiao N, Zhou S, Lin K, Wang D (2015). Malaria imported from Ghana by returning gold miners, China, 2013. Emerg Infect Dis.

[27] Suliman MMA, Hamad BM, Albasheer MMA, Elhadi M, Amin Mustafa M, Elobied M (2016). Molecular evidence of high proportion of *Plasmodium vivax* malaria infection in White Nile area in Sudan. J Parasitol Res..

[28] Snounou G, Viriyakosol S, Jarra W, Thaithong S, Brown KN (1993). Identification of the four human malaria parasite species in field samples by the polymerase chain reaction and detection of a high prevalence of mixed infections. Mol Biochem Parasitol.

[29] Comeron JM (1995). A method for estimating the numbers of synonymous and nonsynonymous substitutions per site. J Mol Evol.

[30] Tajima F (1989). Statistical method for testing the neutral mutation hypothesis by DNA polymorphism. Genetics.

[31] Fu Y-X (1997). Statistical tests of neutrality of mutations against population growth, hitchhiking and background selection. Genetics.

[32] Librado P, Rozas J (2009). DnaSP v5: a software for comprehensive analysis of DNA polymorphism data. Bioinformatics.

[33] Gosi P, Khusmith S, Khalambaheti T, Lanar DE, Schaecher KE, Fukuda MM (2008). Polymorphism patterns in Duffy-binding protein among Thai *Plasmodium vivax* isolates. Malar J..

[34] Ju H-L, Kang J-M, Moon S-U, Kim J-Y, Lee H-W, Lin K (2012). Genetic polymorphism and natural selection of Duffy binding protein of *Plasmodium vivax* Myanmar isolates. Malar J..

[35] Premaratne PH, Aravinda BR, Escalante AA, Udagama PV (2011). Genetic diversity of *Plasmodium vivax* Duffy binding protein II (PvDBPII) under unstable transmission and low intensity malaria in Sri Lanka. Infect Genet Evol..

[36] González-Cerón L, Cerritos R, Corzo-Mancilla J, Santillán F (2015). Diversity and evolutionary genetics of the three major *Plasmodium vivax* merozoite genes participating in reticulocyte invasion in southern Mexico. Parasit Vectors..

[37] Sousa TN, Tarazona-Santos EM, Wilson DJ, Madureira AP, Falcao PRK, Fontes CJF (2010). Genetic variability and natural selection at the ligand domain of the Duffy binding protein in brazilian *Plasmodium vivax* populations. Malar J..

[38] Gunalan K, Lo E, Hostetler JB, Yewhalaw D, Mu J, Neafsey DE (2016). Role of *Plasmodium vivax* Duffy-binding protein 1 in invasion of Duffy-null Africans. Proc Natl Acad Sci USA.

[39] Hans D, Pattnaik P, Bhattacharyya A, Shakri AR, Yazdani SS, Sharma M (2005). Mapping binding residues in the *Plasmodium vivax* domain that binds Duffy antigen during red cell invasion. Mol Microbiol.

[40] McHenry AM, Barnes SJ, Ntumngia FB, King CL, Adams JH (2011). Determination of the molecular basis for a limited dimorphism, N417K, in the *Plasmodium vivax* Duffy-binding protein. PLoS One.

[41] VanBuskirk KM, Sevova E, Adams JH (2004). Conserved residues in the *Plasmodium vivax* Duffy-binding protein ligand domain are critical for erythrocyte receptor recognition. Proc Natl Acad Sci USA.

[42] Babaeekho L, Zakeri S, Djadid ND (2009). Genetic mapping of the duffy binding protein (DBP) ligand domain of *Plasmodium vivax* from unstable malaria region in the Middle East. Am J Trop Med Hyg.

[43] Coniglio ND, Hoxhaj R, Seric A (2017). The demand for foreign workers by foreign firms: evidence from Africa. Rev World Econ..

[44] Payne RO, Silk SE, Elias SC, Milne KH, Rawlinson TA, Llewellyn D (2017). Human vaccination against Plasmodium vivax Duffy-binding protein induces strain-transcending antibodies. JCI Insight..

