# Supplementary figures and images for: Diversity pattern of Duffy binding protein sequence among Duffy-negatives and Duffy-positives in Sudan
Source: Malar J. 2018 Aug 17;17:297. doi: 10.1186/s12936-018-2425-z (PMC6098642; doi:10.1186/s12936-018-2425-z)

**Additional file 1.** Map of Sudan indicating the sample collection sites

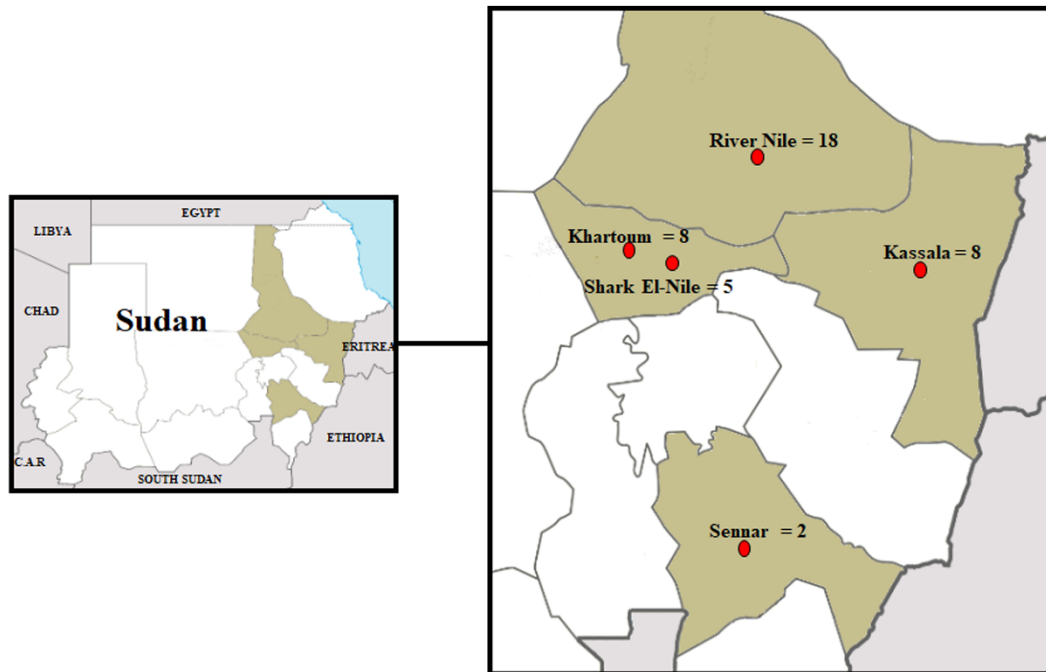

Supplement: Supplementary file 1 — Additional file 1. Map of Sudan indicating the sample collection sites. [file 12936_2018_2425_MOESM1_ESM.pdf]
