# Supplementary material for: Diversity pattern of Duffy binding protein sequence among Duffy-negatives and Duffy-positives in Sudan
Source: Malar J. 2018 Aug 17;17:297. doi: 10.1186/s12936-018-2425-z (PMC6098642; doi:10.1186/s12936-018-2425-z)
Supplement: Supplementary file 2 — Additional file 2. Haplotypes based on 10 common polymorphism sites in Sudan isolates. [file 12936_2018_2425_MOESM2_ESM.pdf]

Additional file 2. Haplotypes based on 10 common polymorphism sites in Sudan isolates

Haplotype in Fig 1A	SNPs on 10 AA positions[a]	Total	Percentage	Code in previous study[b]
Sal-1	G A A G G G T T T T	0	0	12
1	G A A G G G A A C A	11	26.19	4
2	T G G A T G A A C A	7	16.67	70
3, 5, 11	G G G G G G A A C A	12	28.57	43
4	G A G G G A T A T T	5	11.90	22
6, 10	G A G G G A T T T A	4	9.52	23
7	G A G G G A T T T T	1	2.38	24
8	G G G A T A A A C A	1	2.38	33
9	G A G G G G A A C A	1	2.38	25

[a] Ten non-synonymous single nucleotide polymorphisms: nt 924 (R308S), 1111 (K371E), 1151 (G384D), 1153 (E385K), 1158 (K386N), 1169 (H390R), 1251 (N417K), 1270 (L424I), 1309 (W437R), and 1508 (I503K), [b] [22]. Note that haplotypes 5 and 11, and 10 correspond to haplotypes 3 and 6, respectively, because 10 non-synonymous SNPs out of 14 non-synonymous SNPs (Fig 1A) were considered in this analysis.
